# Acute Rejection With DSA‐Negative Severe Microvascular Inflammation in a Kidney Transplant Recipient With an Isolated DPB1*04‐Mismatch Successfully Stabilised With Daratumumab

**DOI:** 10.1111/tan.70560

**Published:** 2026-01-09

**Authors:** Laura Knödl, Maike Büttner‐Herold, Markus Götz, Markus Luber, Bernd Spriewald, Michael Oellerich, Julia Beck, Bernhard Banas, Daniel Zecher

**Affiliations:** ^1^ Department of Nephrology University Hospital Regensburg Regensburg Germany; ^2^ Department of Nephropathology Institute of Pathology, Friedrich‐Alexander‐Universität Erlangen‐Nuremberg (FAU) and University Hospital Erlangen Germany; ^3^ Department of Surgery University Hospital Regensburg Regensburg Germany; ^4^ Department of Internal Medicine 5‐Hematology and Oncology University Hospital Erlangen, Friedrich‐Alexander University Erlangen‐Nürnberg Erlangen Germany; ^5^ Department of Clinical Pharmacology University Medical Center Göttingen Göttingen Germany; ^6^ Chronix Biomedical GmbH Göttingen Germany

**Keywords:** antibody‐mediated rejection, daratumumab, microvascular inflammation

## Abstract

Microvascular inflammation (MVI) in kidney allografts in the absence of detectable donor‐specific anti‐HLA antibodies (DSA) is increasingly recognised as a cause of premature graft failure following kidney transplantation. Potential mechanisms include NK cell alloreactivity mediated by recognition of mismatched HLA class I molecules (missing‐self) via killer‐immunoglobulin‐like receptors. Here, we report the case of an early kidney allograft rejection with severe MVI on biopsy in a patient that was fully HLA‐matched except for a HLA‐DPB1*04 mismatch in the donor. There were no detectable DSA at any time. MVI was successfully reversed and clinically stabilised with a 9‐month course of daratumumab (anti‐CD38 mAb). This case suggests alternative mechanisms of alloreactivity, such as NK cell‐mediated effects, and highlights the existence of MVI in the absence of detectable B cell alloreactivity. Moreover, this case exemplifies the potential of anti‐CD38 treatment in these patients.

## 1 Introduction

Antibody‐mediated rejection (AMR), histologically characterised by microvascular inflammation (MVI) in glomeruli and peritubular capillaries, is one of the most common causes of premature kidney allograft loss [1]. Several observational studies have highlighted the existence of MVI in the absence of detectable donor‐specific anti‐HLA antibodies (DSA) [2], raising strong interest in deciphering alternative mechanisms of alloreactivity in addition to T‐ and B cell‐mediated graft injury [3]. NK cells have recently emerged as a potent mediator of MVI [4, 5], strongly supported by clinical data showing that NK cell depletion by anti‐CD38 treatment can resolve MVI in the context of AMR [6]. NK cells recognise ‘missing self’, that is, the absence of own HLA molecules on donor cells, primarily via killer‐immunoglobulin‐like receptors (KIR). ‘Missing self’ has been traditionally linked to the expression of HLA class I molecules [7]. Herein, we present the case of an early severe and persistent kidney allograft rejection in a recipient with no detectable DSA exclusively mismatched in HLA‐DPB1, suggesting alternative and clinically relevant mechanisms of alloreactivity.

## 2 Materials and Methods

### 2.1 Treatment Protocol and Immunosuppression

Daratumumab was administered intravenously at a dose of 16 mg/kg body weight. Initially, the antibody was given weekly for 8 weeks, following every other week (16 weeks) and every 4 weeks thereafter [8]. The patient was notified about the off‐label use of daratumumab and gave written informed consent. Throughout daratumumab treatment, the patient was kept on triple immunosuppression with tacrolimus, mycophenolate‐mofetil, and prednisolone. Following the diagnosis of AMR, tacrolimus trough levels were kept between 8 and 10 μg/L until month 12 post‐transplantation and between 6 and 8 μg/L thereafter. Mycophenolate‐mofetil was kept at the maximum tolerable dose. Prednisolone was kept at 5 mg qd.

### 2.2 Renal Biopsies

Kidney biopsies were classified according to the Banff 2022 scheme [9]. The follow‐up biopsy 9 months after treatment initiation with daratumumab was also examined using the Molecular Microscope Diagnostic System (MMDx). Therefore, kidney biopsy tissue is analysed by measuring gene expression patterns using microarray technology. mRNA extracted from biopsy tissue hybridises to probes representing key transplant‐related genes. Machine learning algorithms compare these molecular profiles to a reference set of previously characterised biopsies to classify transplant rejection phenotypes such as T cell‐mediated rejection (TCMR) and antibody‐mediated rejection (AMR). In addition, scores for parenchymal injury, including acute kidney injury (AKI), irreversible atrophy and fibrosis, and probability of graft survival are calculated [10].

### 2.3 HLA‐Typing and HLA‐Antibody Detection

High‐resolution HLA typing of donor and recipient was performed according to the standards of the European Federation for Immunogenetics (EFI) using a CE‐certified NGS typing assay (MiaFora MFlex 11, SR‐800‐10534; Werfen) following the manufacturer's instructions. Sequencing was carried out on a MiSeq device (Illumina). Antibody testing was performed according to EFI standards. Anti‐HLA antibody detection was carried out using a screening assay for both HLA class I and II (Labscreen Mixed Class I + II, LSM12; One Lambda). The anti‐HLA antibody specificity of sera was determined using a single antigen assay for HLA class I (i.e., HLA‐A/B/C; LABScreen Single Antigen Assay, LS1A04; One Lambda) and HLA class II antigens (i.e., HLA‐DR/DQ/DP; LABScreen Single Antigen Assay, LS2A01; One Lambda). Tests were carried out according to the manufacturer's instructions and analysed on a Luminex Flexmap 3D flow analyzer (Luminex). A positive result for antibody specificities in Single Antigen bead array was defined as a baseline normalised MFI > 500.

### 2.4 Donor‐Derived Cell‐Free DNA

Donor‐derived cell‐free DNA levels (dd‐cfDNA) were determined by droplet digital PCR with preselected SNPs [11].

## 3 Case Report

In May 2023, a 48‐year‐old female with a 4‐year history of end‐stage renal disease due to hypertensive nephropathy received a first kidney transplant at our institution. High resolution (2‐field) HLA typing of donor and recipient revealed a complete HLA match between donor and recipient in HLA‐A, ‐B, ‐C, ‐DR, ‐DQA1, ‐DQB1 and ‐DPA1 with an isolated mismatch in HLA‐DPB1 (Table 1). There were no preformed DSA (Table S1). Induction therapy consisted of basiliximab followed by tacrolimus, mycophenolate mofetil and prednisolone maintenance immunosuppression. Blood type of donor and recipient was O. Cold and warm ischemia times were 17 h and 45 min, respectively. Due to CMV D+/R‐ high risk constellation, prophylaxis with valganciclovir was given for 3 months. From July 2023 on, a low‐level viremia for CMV was detected intermittently with a maximum of 710 copies/ml, without evidence for CMV disease. Throughout the therapy with daratumumab, no relevant CMV viremia was detected.

**TABLE 1:** High‐resolution HLA‐typing of donor and recipient.

HLA locus	Donor	Recipient
A	*01:01, *68:01	*01:01, *68:01
B	*08:01, *44:02	*08:01, *44:02
C	*07:01, *07:04	*07:01, *07:04
DRB1	*03:01, *11:01	*03:01, *11:01
DRB3	*01:01, *02:02	*01:01, *02:02
DQA1	*05:01, *05:01	*05:01, *05:01
DQB1	*02:01, *03:01	*02:01, *03:01
DPA1	*01:03	*01:03, *02:07
**DPB1**	*04:01, *04:02	*02:01, *19:01

*Note:* Bold font is only used to highlight the differences in HLA typing in this locus between donor and recipient. No statistical test applies here.

**FIGURE 1:**
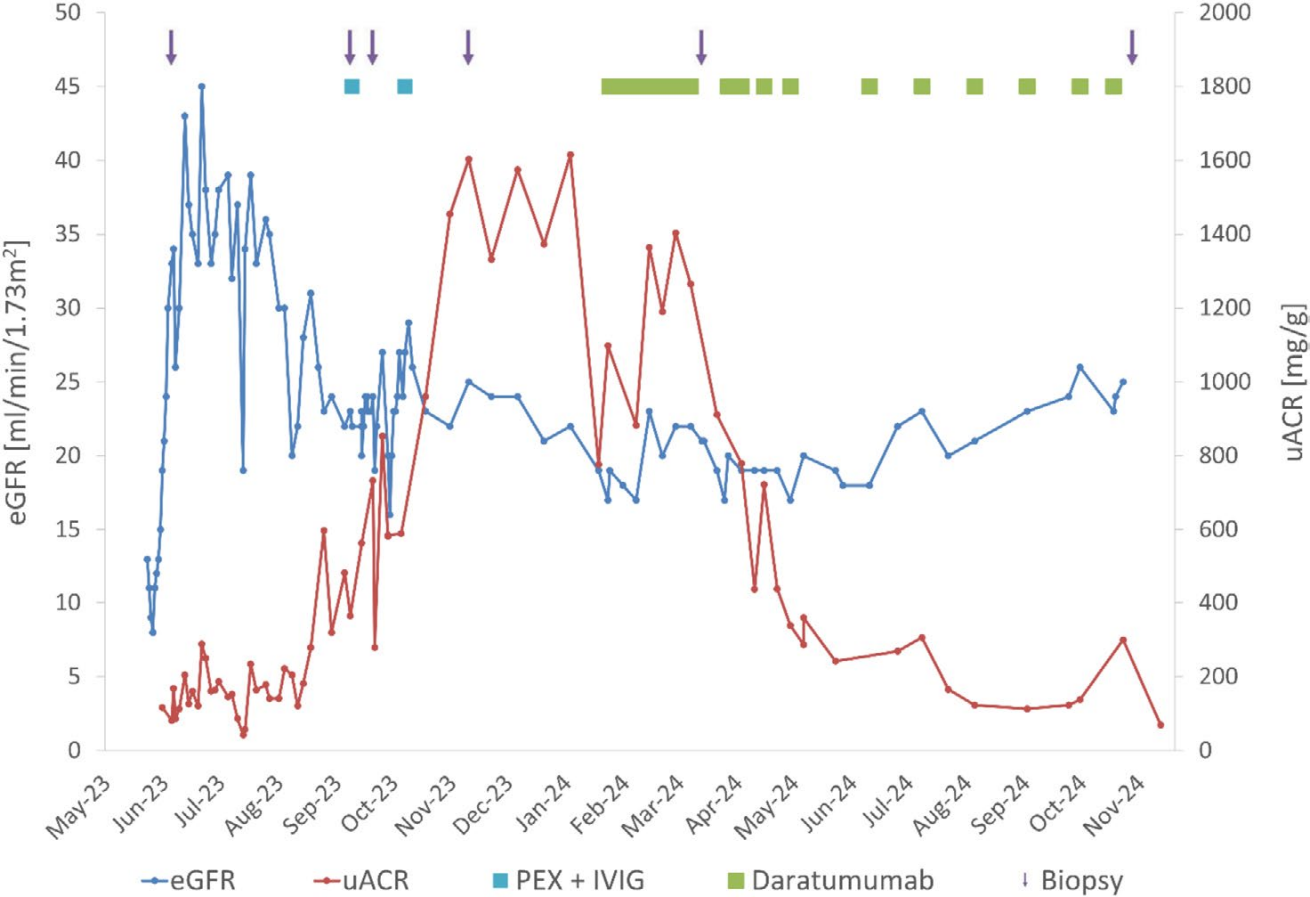
Impact of daratumumab (anti‐CD38) on graft function and albuminuria. Estimated glomerular filtration rate (eGFR in ml/min/1.73m^2^ according to CKD‐EPI) and urinary albumin/creatinine ratio (uACR in mg/g creatinine) during follow‐up. IVIG, intravenous immunoglobulins; PEX, plasma exchange.

Graft function stabilised at an eGFR of 30–40 mL/min/1.73m^2^ (CKD‐EPI) 2 weeks after surgery when a first protocol biopsy revealed isolated glomerulitis (Table S2), that was not treated. Following several episodes of urinary tract infections, eGFR deteriorated to 20 mL/min/1.73m^2^ over the next months. In September 2023, another biopsy now showed severe C4d‐positive MVI with glomerulitis (g) and peritubular capillaritis (ptc) together with borderline changes (Banff lesion score: g3 ptc2 cg0 t1 i1 ti1 v0 ct1 cv1 aah3, C4d1) and 10% interstitial fibrosis and tubular atrophy (IFTA) (Table S2). Light microscopy showed no evidence for thrombotic microangiopathy (TMA, Figure S1A) and immunohistochemistry was minimally positive for C4d in the peritubular capillaries (Figure S1B). Luminex single antigen bead (SAB) testing was negative for DSA. In addition, non‐HLA antibodies (AT1R‐, Endothelin‐Receptor‐ and MICA‐Abs) were undetectable. Nevertheless, treatment with plasma exchange and intravenous immunoglobulins resulted in stabilisation of graft function (Figure 1). A biopsy 4 weeks after treatment revealed complete resolution of MVI but progression of IFTA to 20% (Table S2).

**FIGURE 2:**
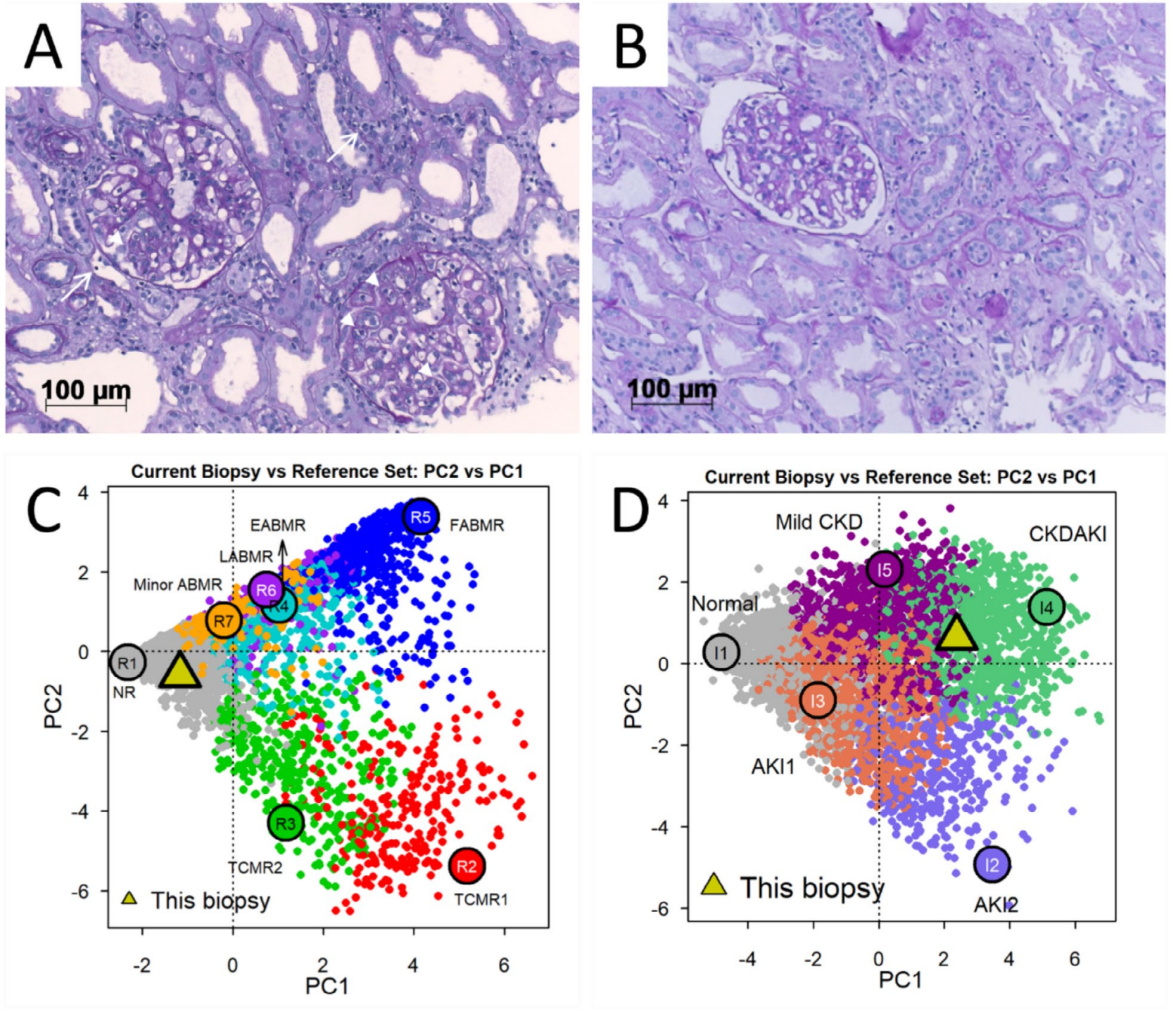
Histological and molecular resolution of microvascular inflammation on kidney biopsies before and after daratumumab treatment. Light microscopy before (A, biopsy #4 in Table S2) and after a 9 months course of daratumumab (B, biopsy #5 in Table S2) revealed glomerulitis (arrow heads) and peritubular capillaritis (arrows) in (A) that was absent in (B) (Periodic acid‐Schiff reaction, original magnification × 200). Molecular analysis after daratumumab treatment shown in principal component plots (PC2 vs. PC1) in relation to a set of reference biopsies (10). (C) The biopsy is classified as no rejection (yellow triangle). The coloured circles indicate archetype rejection phenotypes (R1: non‐rejecting; R2: TCMR 1; R3: TCMR 2; R4: early‐stage ABMR; R5: fully developed ABMR; R6: late‐stage ABMR; R7: minor ABMR). (D) Molecular analysis of injury archetypes indicates chronic kidney damage (CKD). The coloured circles indicate injury phenotypes (I1: normal; I2: AKI2; I3: AKI1; I4: CKDAKI; I5: mild CKD). ABMR, antibody‐mediated rejection; AKI, acute kidney injury; TCMR, T cell‐mediated rejection.

In November 2023, a marked increase in albuminuria (1.7 g/g creatinine) triggered another biopsy showing recurrence of MVI (g2, ptc1) without C4d‐deposition but signs of chronic transplant glomerulopathy (cg2) (Figure 2A and Table S2). Luminex SAB testing was repeatedly negative for DSA during follow‐up (Table S1). After approval by the patient's health insurance, treatment with daratumumab was initiated in January 2024 following a protocol for multiple myeloma at a dose of 16 mg/kg body weight over 9 months. Daratumumab treatment resulted in a profound decrease in albuminuria and stabilisation of allograft function (Figure 1). Follow‐up biopsies 2 and 9 months after treatment initiation revealed complete resolution of MVI (Figure 2B and Table S2). Molecular analysis of the last biopsy revealed low molecular scores for overall rejection (0.24, range 0.0–1.0, upper limit of normal 0.30), T‐cell mediated rejection (TCMR) (0.06, range 0.0–1.0, upper limit of normal 0.10) and antibody‐mediated rejection (ABMR) (0.09, range 0.0–1.0, upper limit of normal 0.20, Figure 2C). The overall inflammation score was moderately increased (1.46, range −3.9 to 5.5, upper limit of normal −0.93) with a markedly elevated fibrosis score (0.8, range 0.0–1.0, upper limit of normal 0.30, Figure 2D), the latter consistent with the histomorphologic findings. Donor‐derived cell‐free DNA levels (dd‐cfDNA) were within normal limits (7 cp/mL; 0.1% of total cell‐free DNA), indicating no ongoing graft injury [12]. Daratumumab treatment was stopped in October 2024. At the last clinical follow up in August 2025, eGFR was stable at 22 mL/min/1.73m^2^ (CKD‐EPI) and albuminuria was low at 66 mg/g creatinine. Dd‐cfDNA levels remained low throughout follow‐up (not shown).

## 4 Discussion

We report on a kidney transplant patient with an isolated HLA‐DPB1*04‐mismatch in the donor that unexpectedly developed fulminant early rejection with severe MVI in the absence of detectable DSA. Daratumumab treatment resulted in histological and molecular resolution of MVI and stabilisation of graft function. Our case is remarkable for several reasons. First, our patient was prioritised within the Eurotransplant Kidney Allocation System due to a ‘full‐house’ match in HLA‐A, ‐B and ‐DR but had early clinical rejection. Second, our case highlights the fluctuating nature of allograft pathology. Most biopsies were classified as C4d‐negative, DSA‐negative MVI as recently defined by the Banff 2022 classification [13]. Despite repeat Luminex SAB testing both before and after transplantation using a low MFI cutoff for positivity (500), we were unable to detect DSA against DPB1*04. Given the C4d‐positivity in the second kidney biopsy, we cannot rule out the existence of subthreshold antibody levels targeting the graft endothelium as recently proposed to underlie DSA‐negative C4d‐positive AMR [14]. Although there is a statistically significant relation between DSA‐positivity and C4d deposition, a causal relationship has never been proven [9]. Other potential causes for C4‐positivity such as TMA [15] were histologically absent. Third, given the near complete HLA match, our case suggests alternative mechanisms of alloreactivity. Of note, the complete HLA class I match between donor and recipient also excludes classic ‘missing‐self’ recognition by NK cells via KIR. Recently, Böhmig et al. reported a statistically significant correlation between various NK cell‐associated gene polymorphisms and MVI independent of DSA [4]. As we did not explore these polymorphisms, we cannot rule out a role for, for example, the C‐type lectin NKG2C receptor or other, so far unknown, innate allorecognition molecules [3, 16]. Interestingly, the concept of ‘missing‐self’ recognition involving exclusively HLA class I was recently challenged by a paper reporting on potent NK cell‐mediated cytotoxicity towards HLA‐DPB1*04:01 via the natural cytotoxicity receptor NKp44 [17], the former being the only mismatched HLA in our patient. NKp44 is only expressed on the cell surface upon activation. NK cells might have been preactivated during the early episodes of urinary tract and subclinical CMV infection [18] in our patient, lending support to the concept of trained innate immunity [19]. Although speculative at this time, exploring the clinical relevance of isolated DPB1*04:01 mismatches might deserve further investigation. Finally, to our knowledge, this is the first reported case of DSA‐negative MVI with a clinical, histological and molecular response to a 9 months course of daratumumab, highlighting the potential of anti‐CD38 treatment strategies even in patients without detectable DSA.

## Author Contributions

L.K., M.G., B.B. and D.Z. were responsible for the clinical treatment of the patient. M.B.‐H., M.L., B.S., M.O. and J.B. performed data analysis and contributed to the writing of the manuscript. L.K. and D.Z. wrote the manuscript.

## Funding

The authors have nothing to report.

## Consent

Informed consent was obtained from the patient for off‐label use of daratumumab and publication of this case report. All efforts were made to ensure patient anonymity.

## Conflicts of Interest

The authors declare no conflicts of interest.

## Supporting information


**Table S1:** Results of Luminex single antigen bead (SAB) testing in chronological order.
**Table S2:** Kidney biopsy results as retrieved from the original reports.
**Figure S1:** Histology of Biopsy #2 with diagnosis of C4d‐positive AMR. (A) Light microscopy shows MVI (g3, ptc2).

## Data Availability

The data that supports the findings of this study are available in Supporting Information of this article.
