# Supplementary material for: Acute Rejection With DSA‐Negative Severe Microvascular Inflammation in a Kidney Transplant Recipient With an Isolated DPB1*04‐Mismatch Successfully Stabilised With Daratumumab
Source: HLA. 2026 Jan 9;107(1):e70560. doi: 10.1111/tan.70560 (PMC12789712; doi:10.1111/tan.70560)

Supplementary Information

Supplementary Table 1: Results of Luminex single antigen bead (SAB) testing in chronological order.

**Date**		**Luminex Single Antigen [MFI]**
Oct 2021	HLA class I	A11[1260] A24[561] A68[900] A69[541] B37[598] B46[13550] B57[1108] B58[550] B60[527] B62[696] B72[621] C2[573] C4[583] C7[611] C9[15039] C10[12988] C12[780] C15[647] C17[1352]
	HLA class II	DR7[4402] DR9[592] DR53[2444] DQ4[527] DQ8[508] DP1[560] DP3[691] DP13[518]
Oct 2022	HLA class I	A11[922] B46[12138] B57[571] B62[554] C9[15008] C10[11704] C17[1092]
	HLA class II	DR7[4849] DR9[591] DR53[3134] DP3[526]
**Post Tx**		
Jun 2023	HLA class I	A11[774] A24[647] B46[6759] B57[538] C9[8460] C10[6061]
	HLA class II	DR7[1799] DR53[1131]
Aug 2023	HLA class I	B46[6098] C9[8091] C10[5230]
	HLA class II	DR7[1640] DR53[1277]
Nov 2023	HLA class I	A11[8348] A30[2543] A34[6068] A66[7110] A68[1204] A69[2312]
	HLA class II	negative
Mar 2024	HLA class I	B46[11386] B51[1925] B57[4234] B62[3873] B71[2451] B72[3981] B75[1813] B77[2338] C9[16033] C10[14369] C15[1681]
	HLA class II	DR7[10132] DR9[1285] DR53[6984]
May 2024	HLA class I	B46[6629] B57[1508] B62[1304] B71[1003] B72[1347] C9[10856] C10[9232]
	HLA class II	DR7[4464] DR53[2677]
Jan 2025	HLA class I	B46[2809] C9[4668] C10[3731]
	HLA class II	negative
Apr 2025	HLA class I	negative
	HLA class II	negative
May 2025	HLA class I	negative
	HLA class II	negative

Supplementary Table 2: Kidney biopsy results as retrieved from the original reports.

	Biopsy #1 06/23	Biopsy #2 09/23	Biopsy #3 09/23	Biopsy #4 11/23	Biopsy #5 03/24	Biopsy #6 10/24
	2-week-protocol	3-month-protocol	post IVIG + PEX	Indication biopsy	2 months dara	9 months dara
**Banff lesion score (2022)**						
- g	2	3	0	2	0	0
- ptc	0	2	0	1	0	0
- t	0	1	1	1	1	1
- i	0	1	1	0	2	0
- ti	1	1	1	1	2	2
- v	0	0	0	0	0	0
- cg	0	0	0	2	0	0
- cv	0	1	1	1	1	1
- ct	1	1	1	1	1	1
- ci	1	1	1	1	1	1
- aah	0	3	0	2	1	2
- i-IFTA	0	1	1	2	1	3
- t-IFTA	0	1	1	1	0	2
- C4d	0	1	0	0	0	0
IFTA	5 %	10 %	20 %	10 %	15 %	20-25 %
AMR (Banff 2022)	MVI, DSA neg, C4d neg	AMR	none	MVI, DSA neg, C4d neg	none	none
TCMR (Banff 2022)	none	borderline	borderline	none	borderline	none

g, glomerulitis; ptc, peritubular capillaritis; t, tubulitis; i, interstitial inflammation; ti, tubulointerstitial inflammation; v, arterial inflammation; cg, transplant glomerulopathy; cv, arterial intimal thickening; ci, interstitial fibrosis; ct, tubular atrophy; aah, arteriolar hyalinosis; IFTA: interstitial fibrosis and tubular atrophy; i-IFTA, interstitial inflammation in IFTA; t-IFTA, tubulitis in IFTA; C4d, C4d-deposition on peritubular capillaries; AMR, antibody-mediated rejection; TCMR, T cell-mediated rejection.

Supplementary Figure 1: Histology of Biopsy #2 with diagnosis of C4d-positive AMR. (A) Light microscopy shows MVI (g3, ptc2) without evidence of thrombotic microangiopathy (Periodic acid-Schiff reaction, original magnification x 200). (B) Immunohistochemistry was minimally positive for C4d in the peritubular capillaries with negative staining in the glomerular capillaries (not shown, original magnification x 200).

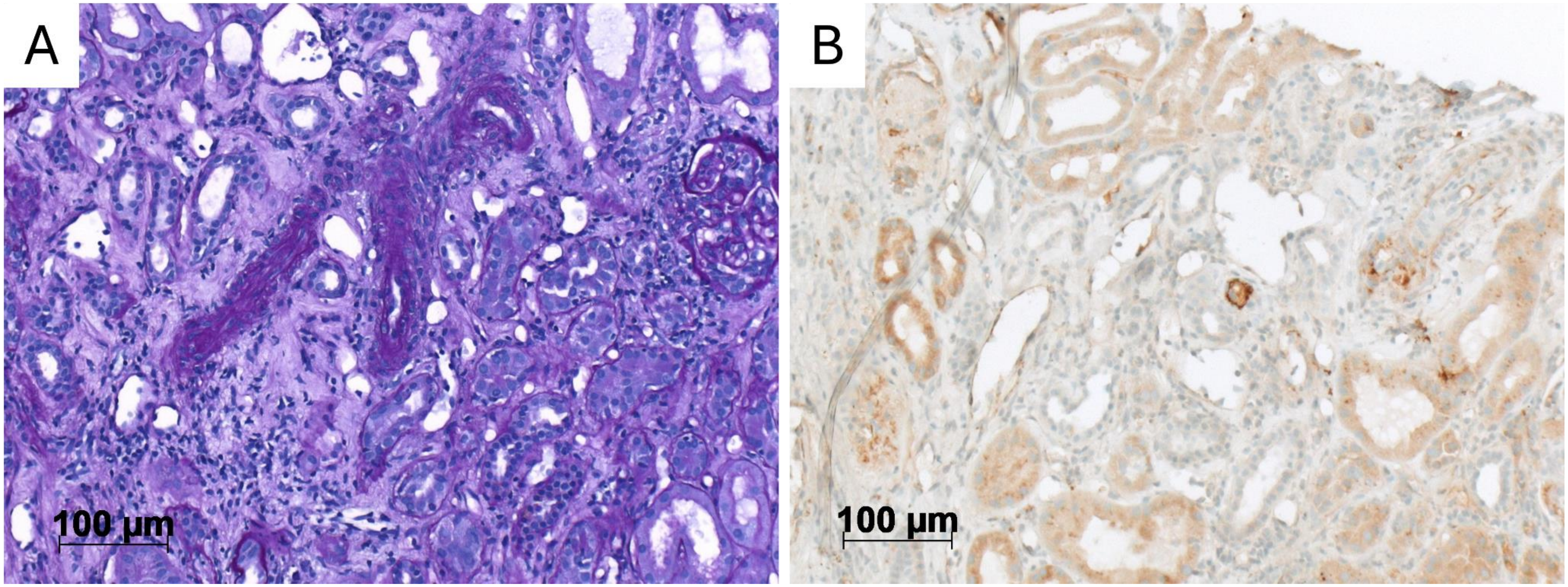

Supplement: Supplementary file 1 — Table S1: Results of Luminex single antigen bead (SAB) testing in chronological order. Table S2: Kidney biopsy results as retrieved from the original reports. Figure S1: Histology of Biopsy #2 with diagnosis of C4d‐positive AMR. (A) Light microscopy shows MVI (g3, ptc2). [file TAN-107-e70560-s001.pdf]
